# The Cost Efficiency of Home Modifications to Reduce Health Care Costs

**DOI:** 10.1093/geroni/igab046.2144

**Published:** 2021-12-17

**Authors:** Jesse Abraham

**Affiliations:** HomesRenewed, Washington, District of Columbia, United States

## Abstract

The existing quantity of housing dedicated for older adults is not sufficient to meet the needs of this growing population. And even as the Centers for Medicare and Medicaid Services reimbursement structures are shifting from traditional inpatient and outpatient settings to care in the home, it is a commonplace that most homes were not designed or built to support the needs of aging residents or the provision of healthcare. It is time for America’s 100 million existing houses to be made as safe and accessible as possible for aging in place. Falls cost over $50 billion a year in medical expenses. This paper distills current knowledge regarding healthcare cost reductions from home modifications, and then calculates the cost efficiency to society and to the federal government of providing government subsidies for home modifications for older adults at the ages of 50, 65 and 75. Cost sharing among insurers, government and the beneficiary is one way to achieve the positive social returns.

